# A selective androgen receptor modulator SARM‐2f activates androgen receptor, increases lean body mass, and suppresses blood lipid levels in cynomolgus monkeys

**DOI:** 10.1002/prp2.563

**Published:** 2020-02-07

**Authors:** Megumi Morimoto, Masuo Yamaoka, Takahito Hara

**Affiliations:** ^1^ Oncology Drug Discovery Unit Pharmaceutical Research Division Takeda Pharmaceutical Company Limited Kanagawa Japan

**Keywords:** body weight, cholesterol, lean body mass, monkey, sarcopenia, selective androgen receptor modulator, testosterone

## Abstract

SARM‐2f a selective androgen receptor (AR) modulator, increases skeletal muscle mass and locomotor activity in rats. This study aimed to clarify its pharmacological effects in monkeys. In reporter assays, the EC_50_ values of SARM‐2f for rat, monkey, and human AR were 2.5, 3, and 3.6 nmol/L, respectively; those of testosterone were 12, 3.2, and 11 nmol/L, respectively. A single oral administration (10 mg/kg SARM‐2f) produced a maximal plasma concentration of 3011 ng/mL, with an area under the 24 hours concentration‐time curve of 8152 ng·h/mL in monkeys. Body weight (BW), lean body mass (LBM), and plasma levels of total cholesterol, triglyceride, high‐density lipoprotein cholesterol, low‐density lipoprotein cholesterol, lipoprotein (a), alanine aminotransferase, and asparagine aminotransferase were measured after 4 weeks of treatment with SARM‐2f (1, 3, and 10 mg/kg/day, QD, p.o.) or testosterone enanthate (TE; 2 mg/kg/2 weeks, s.c.) in monkeys. BW and LBM were significantly increased by 12% each by SARM‐2f at 10 mg/kg, and by 5% and 8%, respectively, by TE, but these effects were not statistically significant. Plasma levels of all lipids were either decreased or showed a tendency to be decreased by SARM‐2f. TE decreased the triglyceride level and increased the low‐density lipoprotein cholesterol level. Liver marker levels were not changed by either SARM‐2f or TE. Our data demonstrated that SARM‐2f exerted anabolic effects and produced a lipid profile that differed from that produced by testosterone in monkeys, suggesting that SARM‐2f might be useful for diseases such as sarcopenia.

## 1 INTRODUCTION

Age‐related sarcopenia or cachexia is associated with a decrease in muscle mass and strength; this reduces the quality of life and increases nursing care requirements and mortality.^1^, ^2^, ^3^ Sarcopenia and cachexia represent unmet needs, and the sarcopenia medical care guidelines were established recently.^4^, ^5^ Medication provides one treatment option, and the ghrelin agonist anamorelin was approved for this condition in the USA in 2017. Anamorelin increased body weight and muscle mass and improved anorexia in cancer cachexia.^6^ However, anamorelin did not improve grip strength.^6^ In contrast, testosterone can improve muscle strength.^7^ However, there is a concern that long‐term testosterone use may cause adverse cardiovascular events because of its impact on the blood lipid profile.^8^, ^9^, ^10^ Thus, the development of alternative therapeutic agents is required.

Selective androgen receptor modulator (SARM) compounds, such as ostarine, significantly increased body weight, muscle mass, and muscle strength in preclinical rodent studies,^11^, ^12^, ^13^ and increased muscle mass in clinical trials.^14^ Our non‐steroidal small molecule, SARM compound, SARM‐2f (4′‐[(2S,3S)‐2‐Ethyl‐3‐hydroxy‐5‐oxopyrrolidin‐1‐yl]‐2′‐(trifluoromethyl) benzonitrile), also increases muscle weight and stimulates motor activity, food intake, and sexual behavior in rodent models.^15^, ^16^, ^17^ Currently, there are only a few reports showing the pharmacological effect of SARM compounds in monkeys: effects on blood high‐density lipoprotein cholesterol (HDL‐c) 18 and overall gene expression profiles in the muscle.^19^ In this study, we investigated the pharmacological effects of SARM‐2f in cynomolgus monkeys in vitro and in vivo. The androgen receptor (AR) activation of monkey AR was examined by reporter assays, the anabolic effects were assessed using body weight (BW) and lean body mass (LBM), and the potential cardiovascular or liver side effects were assessed by blood biochemistry tests. This study shows the anabolic effect of a SARM compound, SARM‐2f, together with its favorable effect on the blood lipid profile in monkeys.

## 2 MATERIALS AND METHODS

### 2.1 Reagents

SARM‐2f (Lot No. B23444‐084‐10) was synthesized by Takeda Pharmaceutical Company Limited. Ostarine was purchased from Cayman Chemical Company. Testosterone and dihydrotestosterone (DHT) were purchased from TCI Chemicals (Tokyo, Japan). Testosterone enanthate (TE; ENARMON DEPOT Intramuscular injection 125 mg) was purchased from Asuka Pharmaceutical. Methylcellulose and sesame oil were purchased from Shin‐Etsu Chemical and Maruishi Pharmaceutical, respectively.

### 2.2 AR reporter assays

COS7 cells were transfected with 0.49 μg of either human AR (pcDNA3.1(‐)/hAR), rat AR (pcDNA3.1/rAR), or cynomolgus monkey AR (pcDNA3.3/mfAR), together with 19.5 μg pGL4.36/MMTV‐luc, using Superfect Transfection Reagent (QIAGEN) according to the manufacturer's instructions. Two hours post‐transfection, the cells were plated in 96‐well plates at a density of 2 × 10^4^ cells/well, and cultured at 37°C for 3 hours. SARM‐2f, ostarine, or testosterone were then added to the wells and incubated at 37°C for 24 hours. Luciferase activity was measured using the Steady‐Glo Luciferase assay system (Promega) and an Envision Multimode Plate Reader (PerkinElmer). Compound activity was calculated with 1 µmol/L DHT activity set at 100%.

### 2.3 Animals

All animal studies were performed at SNBL Japan; the animals were maintained in accordance with the rules of the Institutional Animal Care and Use Committee of Takeda Pharmaceutical Company Limited. The animals were purpose‐bred Chinese cynomolgus monkeys. In the single‐dose pharmacokinetic test, two males and two females aged 5‐7 years were used. For the multi‐dose efficacy test, 20 males aged 3‐4 years were purchased and allowed to habituate for two weeks. Fifteen animals were selected based on their weight and appropriateness for administration. The experiments were conducted in an individual breeding environment with a 12 hours light/dark cycle (light period 07:00‐19:00 hours), a temperature of 23°C‐29°C, and 35‐75% humidity. The monkeys received 9 × 12 g pellets of diet (HF Primate 5K91 12G 5K9J) once daily at 14:30‐16:00 hours; this was retrieved the next morning at 08:30‐10:00 hours (before dosing). Fresh apple or sweet potato was provided more than twice a week, except on the day of measuring BW and collecting blood. Water was available ad libitum. Toys were provided continuously.

### 2.4 Pharmacokinetic study

Monkeys were orally treated with SARM‐2f (10 mg/kg) as a single dose or daily administration for 14 consecutive days; blood was collected from the femoral vein at 0.5, 1, 2, 4, 8, and 24 hours after the single dose and 14 doses (n = 2 each sex). The blood samples were centrifuged (4°C, 12 000 g, 1 minutes) to obtain plasma. Plasma concentrations of SARM‐2f were measured by liquid chromatography/tandem mass spectrometry.

### 2.5 Effects on body weight, lean body mass, and blood biochemistry

The monkeys were divided into five groups by weight randomization (MiTOX system ver2.0, Mitsui E & S Systems Research Inc) on the last day of the habituation period. SARM‐2f was orally administered at daily doses of 1, 3, or 10 mg/kg for 4 weeks between 9:00 and 11:00 hours, using 0.5% *w/v* methylcellulose as the vehicle. TE was injected intramuscularly on Day 0 and Day 13; the dosing start was set at Day 0 at a dose of 2 mg/kg using sesame oil as the vehicle. The route of administration and dose of TE was selected according to the route and dose mentioned in the clinical indications. BW was measured every week before dosing (between 8:30 and 9:00 hours), and LBM was measured every 2 weeks after dosing and before feeding (between 11:30 and 14:30 hours) using an X‐ray bone density measuring device (Discovery, HOLOGIC USA). Blood collection for lipid profiles was conducted on the day after the completion of the four‐week multiple administration of SARM‐2f or TE. Plasma levels of total cholesterol (T‐chol), HDL‐c, low‐density lipoprotein cholesterol (LDL‐c), triglyceride (TG), alanine aminotransferase (ALT), and asparagine aminotransferase (AST) were measured by an automatic JCA‐BM8 analysis system (JEOL Ltd.). Plasma lipoprotein (a) (Lp (a)) levels were measured using a multiplate absorbance measuring system (Multiscan Ascent, Thermo Fisher Scientific KK). Plasma testosterone levels were measured using a testosterone measurement kit (TESTO‐CTK, DiaSorin Inc Saluggia Italy).

### 2.6 Statistical analysis

In the reporter assays, EC_50_ was calculated using Graph Pad Prism version 6. The effects of SARM‐2f or TE on BW and LBM were analyzed by *t* test, with Bonferroni correction for repeated measurements. The effects of SARM‐2f on lipid levels and ALT/AST levels were analyzed by Dunnett's test after ANOVA using the SAS software system, and those of testosterone were analyzed by Student's *t* test. *P* < .05 was considered statistically significant.

## 3 RESULTS

### 3.1 Activation of monkey, human, and rat AR by SARM

**Figure 1:**
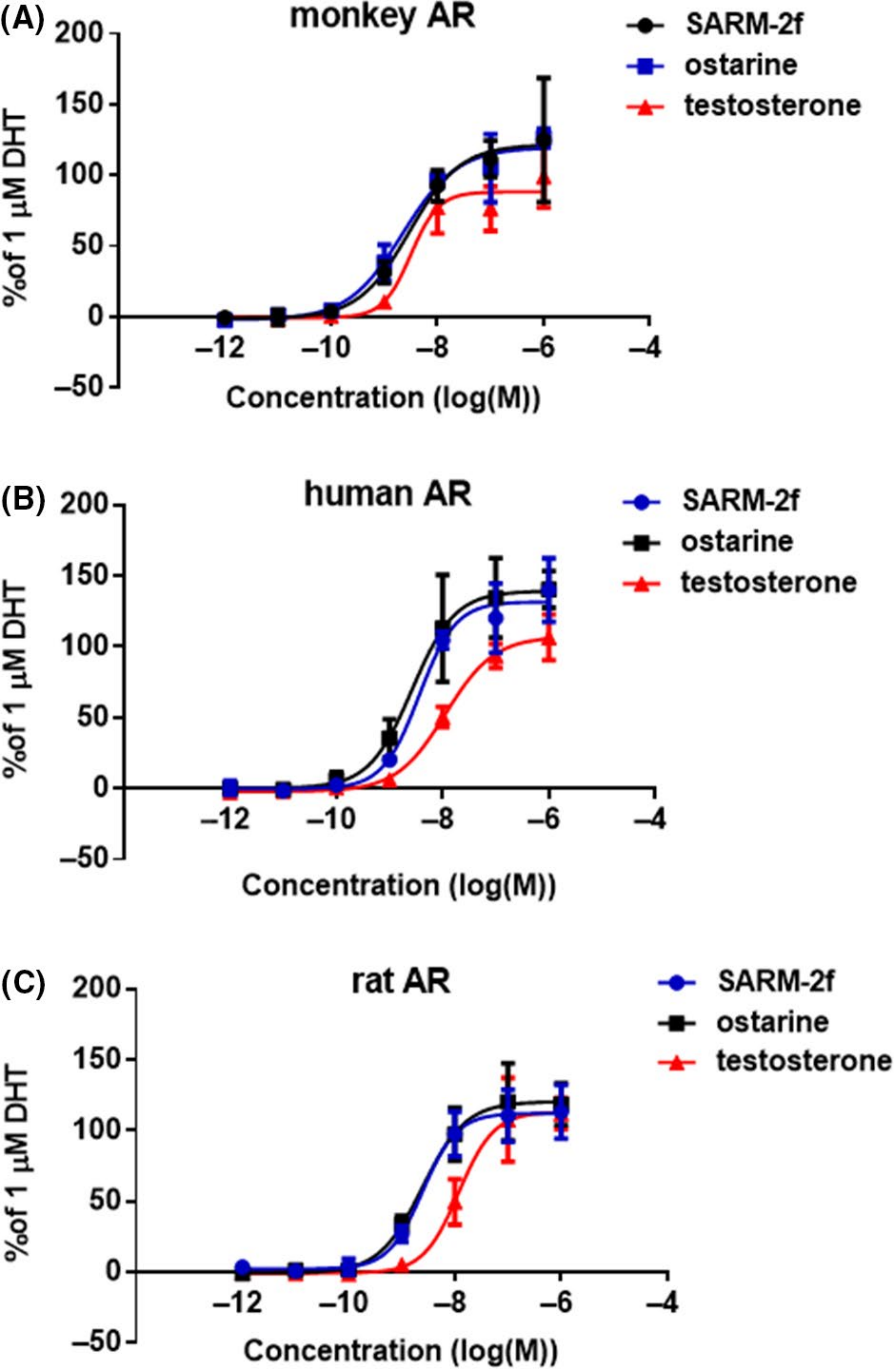
Androgen receptor (AR) transcriptional activity of SARM‐2f, ostarine and testosterone. The reporter assays using COS7 cells expressing (A) monkey AR; (B) human AR; and (C) rat AR were conducted as described in the Methods section. The AR agonistic activities are expressed relative to those of the 1 μmol/L dihydrotestosterone (DHT) treatment as 100%. Luciferase activities for 1 μmol/L DHT in monkey, human and rat ARs were 119 453 ± 19 175 cps, 104 280 ± 9931 cps, and 100 160 ± 8.508 cps, respectively. Data are shown as the mean ± SD (n = 3)

For monkey AR, the dose‐response curves for SARM‐2f, ostarine, and testosterone were similar over the concentration range of 10^−12^ to 10^−8^ mol/L, with respective EC_50_ values of 3 (confidence interval (CI): 1.2‐7.2), 2.2 (CI: 1.2‐4.1), and 3.2 (CI: 1.4‐7.2) nM (Figure 1A). The maximal AR activation was 125% and 126% for SARM‐2f and ostarine at 1 μmol/L, respectively, where the activation by DHT at 1 μmol/L was set to 100% (Figure 1A). The activation by 1 μmol/L testosterone was very similar, at 99% of 1 μmol/L DHT activation. For human AR, the slopes of the SARM‐2f and ostarine dose‐response curves were steeper than that of testosterone, with EC_50_ values of 3.6 (CI: 2.1‐6.2), 2.7 (CI: 1.3‐5.5), and 11 (CI: 7.5‐17) nmol/L, respectively, (Figure 1B). The maximal activation levels were 140%, 141%, and 107% for SARM‐2f, ostarine, and testosterone, respectively, at 1 μmol/L (Figure 1B). For rat AR, the dose‐response curves of SARM‐2f and ostarine shifted to lower concentrations, compared to that of testosterone (Figure 1C), and the EC_50_ values were 2.5 (CI: 1.4‐4.3), 2.5 (CI: 1.3‐4.5), and 12 (CI: 7.1‐20) nM, respectively (Figure 1). The maximal activations were 118, 112, and 113% for SARM‐2f, ostarine, and testosterone, respectively, at 1 μmol/L (Figure1C). A preliminary reporter assay was conducted in a similar manner. Similar results were obtained from a total of two experiments.

### 3.2 Pharmacokinetics of SARM‐2f in monkeys

Following a single oral dose of 10 mg/kg SARM‐2f, the maximal plasma level (*C*_max_) was 3011 ng/mL; this was observed at 1.0 hours (*T*_max_), and the area under the plasma concentration‐time curve (AUC_0‐24h_) was 8152 ng·h/mL (n = 2 each sex, Table 1). The *T*_max_, *C*_max_, and AUC_0‐24h_ observed on the day after the 14 days of daily oral treatments did not differ from those observed after the single oral administration (Table 1). The blood concentration curves are shown in Figure S1.

**Table 1:** Kinetic parameters for SARM‐2f in cynomolgus monkeys

Dose mg/kg/day		Male (N = 2)			Female (N = 2)			Total (N = 4)		
		*T*_max_	*C*_max_	AUC 0‐24 h	*T*_max_	*C*_max_	AUC 0‐24 h	*T*_max_	*C*_max_	AUC 0‐24 h
		(h)	(ng/mL)	(ng·h/mL)	(h)	(ng/mL)	(ng·h/mL)	(h)	(ng/mL)	(ng·h/mL)
10	1st	1.0	2681	8188	1.0	3341	8116	1.0 ± 0.0	3011 ± 718	8152 ± 945
	14th	0.8	3176	8620	1	2297	6043	0.9 ± 0.3	2736 ± 525	7331 ± 1618

Note

Male and female cynomolgus monkeys were treated with SARM‐2 (10 mg/kg) single dose or daily oral administration for 14 consecutive days.

AUC0‐24 h, area under curve from time 0 to 24 h; *C*_max_, peak plasma concentration; *T*_max_, time at *C*_max_.

### 3.3 Increase of BW and LBM during four‐week administration of SARM‐2f to monkeys

**Figure 2:**
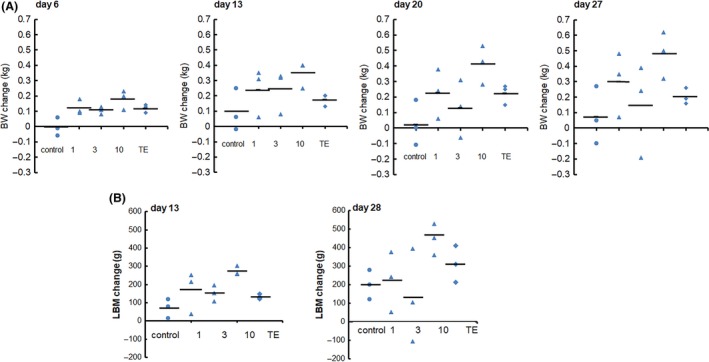
Anabolic effects of SARM‐2f and testosterone enanthate (TE) in monkeys. Male cynomolgus monkeys were treated with daily oral SARM‐2 (1, 3, or 10 mg/kg) or intramuscular injections once every 2 wks of TE (2 mg/kg) for 4 wks. Individual data of (A) body weight (BW) on Days 6, 13, 20, and 27 and (B) lean body mass (LBM) on Days 13 and 28 are shown in the figure. The average value is shown as horizontal lines in each group (n = 3). BW increased significantly on Day 27 by 10 mg/kg SARM‐2f. LBM also showed a significant increase on Day 28 by 10 mg/kg SARM‐2f. TE, as well as 1 and 3 mg/kg SARM‐2f, showed no significant increase of BW and LBM during the study period. *P < .05 by Student's *t* test, with Bonferroni correction for repeated measurements

In the vehicle control animals, the BW gains from the pre‐dosing level (Day −1 when dosing start was set at Day 0) to Day 13 and Day 27 were 2.4% and 1.8%, respectively (Figure 2A, Table S1). In contrast, animals receiving daily doses of SARM‐2f at 1, 3, and 10 mg/kg increased BW by 6.2%, 6.0%, and 8.7%, respectively, on Day 13 (Figure 2A, Table S1), and 7.7%, 3.3%, and 11.9%, respectively, on Day 27 (Figure 2A, Table S1). The BW increase on Days 20 and 27 in animals treated with 10 mg/kg SARM‐2f was statistically significant (Figure 2A). Animals receiving subcutaneous TE at 2 mg/kg were 4.3% and 4.9% heavier on Days 13 and 27, respectively, but these differences were not statistically significant (Figure 2A, Table S1).

LBM increase from the pre‐dosing level to Day 13 and Day 28 of daily vehicle dosing was 2.0% and 5.3%, respectively, (Figure 2B, Table S2). By contrast, animals receiving daily doses of SARM‐2f at 1, 3, and 10 mg/kg showed LBM increases of 4.3%, 4.0%, and 7.1%, respectively, on Day 13 (Figure 2B, Table S2), and 6.1%, 3.1%, and 11.8%, respectively, on Day 28 (Figure 2B, Table S2). The LBM increase in animals treated with 10 mg/kg SARM‐2f was statistically significant on both Days 13 and 28 (Figure 2B, Table S2). TE at 2 mg/kg increased LBM by 3.5% and 8.1% on Days 13 and 28, respectively, (Figure 2B, Table S2), but these were not statistically significant.

### 3.4 Blood chemistry

**Figure 3:**
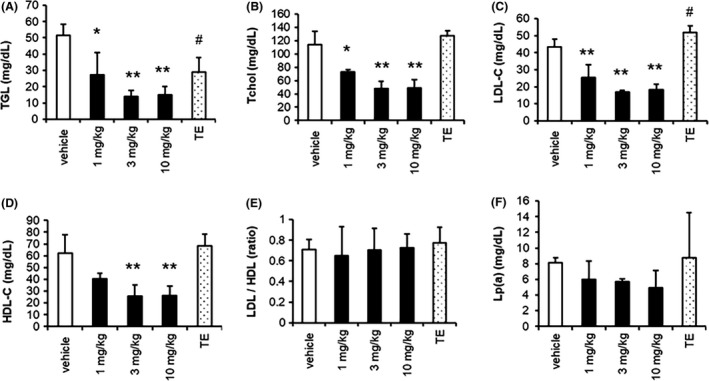
Plasma lipid levels after 4‐wk treatments with SARM‐2f or testosterone enanthate (TE) in monkeys. Male cynomolgus monkeys were treated with daily oral SARM‐2 (1, 3, or 10 mg/kg) or intramuscular injections once every 2 weeks of TE (2 mg/kg) for 4 wks. Plasma levels of (A) triglyceride (TG); (B) total cholesterol(T‐chol); (C) low‐density lipoprotein cholesterol (LDL‐c); (D) high‐density lipoprotein cholesterol (HDL‐c); (E) HDL‐c/LDL‐c ratio; and (F) lipoprotein (a)(Lp(a)) are shown. SARM‐2f significantly reduced the TG, T‐chol, and LDL‐c levels at all doses and reduced the HDL‐c level significantly at 3 and 10 mg/kg. TE significantly reduced the TG level and increased the LDL‐c level. Data represent the mean ± SD(n = 3); The effects of SARM‐2f on lipid levels were analyzed by Dunnett's test, and those of testosterone were analyzed by Student's *t* test. *P < .05, **P < .01 by Dunnett's. ^#^ *P* < .05 by Student's *t* test

**Figure 4:**
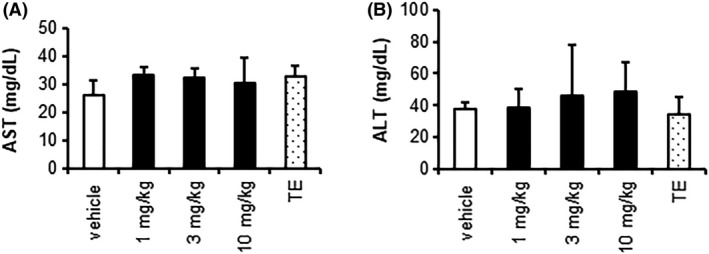
Liver function tests after 4‐wk treatments with SARM‐2f or testosterone enanthate (TE) in monkeys. Male cynomolgus monkeys were treated with daily oral SARM‐2 (1, 3, or 10 mg/kg) or intramuscular injections once every 2 wks of TE (2 mg/kg) for 4 wks. Plasma levels of (A) asparagine aminotransferase (AST); (B) alanine aminotransferase (ALT) are shown. Treatment with SARM‐2f or TE did not alter the ALT and AST levels, either. Data represent the mean ± SD(n = 3); The effects of SARM‐2f on ALT and AST levels were analyzed by Dunnett's test, and those of testosterone were analyzed by Student's *t* test

Blood levels of T‐chol, LDL‐c, and TG were lower following daily treatment with SARM‐2f at 1, 3, and 10 mg/kg (Figure 3A‐C), while the HDL‐c level was only reduced in animals treated with 3 or 10 mg/kg (Figure 3D). SARM‐2f did not cause any significant change in the HDL‐c/LDL‐c ratio (Figure 4E). The Lp (a) protein level showed a tendency to be decreased by SARM‐2f (Figure 3F), but this effect was not statistically significant. TE did not affect the blood levels of T‐chol, HDL‐c, or Lp (a) (Figure 3A,D,F), but did reduce the TG level and increase the LDL‐c level significantly (Figure 3C,B). The blood levels of AST and ALT were not changed by either SARM‐2f or TE (Figure 4A,4).

### 3.5 Plasma testosterone level

Blood testosterone levels were measured before the dosing started (Day −11), and 2 weeks after the second dose (Day 27) in the vehicle and TE groups. In the TE group, all monkeys showed an increase on Day 27, as compared with pre‐dosing levels (Table 2); the average concentration changed from 1.0 ng/mL on Day −11 to 4.2 ng/mL on Day 27 (Table 2). In the vehicle group, one of the three animals showed an increase over time (Table 2); this individual also showed gains in LBM and BW (Figure 2). The testosterone levels of the remaining two animals did not increase during vehicle treatment (Table 2).

**Table 2:** Individual plasma testosterone levels in male cynomolgus monkeys before and after the indicated 4‐week treatments

Treatment	Animal	Testosterone concentration (ng/mL)	
	No.	day‐11	day27
Vehicle control	1	1.06	3.75
	2	0.97	1.24
	3	0.34*	0.36
	Mean	0.79	1.78
	SD	0.39	1.76
Testosterone enanthate	1	1.03	7.00
	2	0.34*	1.78
	3	1.71	3.92
	Mean	1.03	4.23
	SD	0.69	2.62

* Reanalysis values. When samples were measured after 2‐fold dilution and were below the lower limit of quantification (0.3 ng/mL), they were reanalyzed without dilution, and these values were presented.

## 4 DISCUSSION

Sarcopenia is defined as an age‐associated loss in skeletal muscle function and muscle mass. The recent treatment guidelines issued by the International Conference on Sarcopenia and Frailty Research strongly recommend the prescription of resistance‐based physical activity, and conditionally recommend protein supplementation/ protein‐rich diet.^5^ However, they do not recommend anabolic hormone prescription, because currently the evidence supporting this hypothesis is limiting.^5^ They state that although low testosterone levels are associated with higher levels of sarcopenia, their systematic review only identified one quality randomized clinical trial, which investigated the effects of MK‐0773, a SARM, in older female adults with sarcopenia; the trial showed an improvement in LBM, but not in strength or function over six months.^19^ However, they also state that in healthy older people and in people with late onset hypogonadism, enobosarm, another SARM improved LBM and stair climb^20^, ^21^ and that in patients with cancer cachexia, similar but less impressive results were observed.^22^ The possible reasons for inadequate efficacy of the SARMs in the clinical trials could be the characteristics of the SARM compounds; chemical structure‐derived off‐target effects or metabolic instability of the compounds, which might hamper the dose increase or the prolongation of treatment period. SARM‐2f is a novel and potent SARM compound, and we did not detect any off‐target/toxic effects or metabolic instability in our assays. Therefore, we believe that SARM‐2f has the potential to demonstrate adequate efficacy in patients with sarcopenia, cachexia, or late onset hypogonadism.

This study investigated whether SARM‐2f exerted anabolic effects on a non‐human primate to explore its potential use in humans. Currently, the pharmacological effects of SARM compounds have not been well‐characterized in monkeys. It is reported that some SARM compounds reduce blood HDL‐c levels^18^ and LGD2941 has an influence on gene expression profile in muscle in monkeys.^23^ In this study, reporter assays revealed that SARM‐2f and testosterone had similar effects on the monkey AR, whereas SARM‐2f had a higher effect than testosterone on the human AR. Multiple oral dosing for 4 weeks in monkeys revealed that SARM‐2f had more potent anabolic effects than testosterone. Monitoring of plasma testosterone levels revealed that this study employed a suitable dose of TE; an increase of 3.2 ng/mL was observed, which is similar to the level achieved by testosterone therapy in a clinical setting^24^, Androgel® (testosterone gel) 1% NDA21‐015.S012 Physician's Package Insert]. Furthermore, in this monkey study, it is necessary to take into consideration that the BW and LBM increase due to increased food consumption was not counted because, from the animal ethics viewpoint, the amount of food served in this study was fixed to ensure the health of the monkeys. This is done to prevent acute distension and constipation caused by the accumulation of gas in the gastrointestinal tract arising from the rapid ingestion of a large amount of food. In this study, there were many individuals who ate all of the food daily (Table S4). It may have been appropriate to slightly increase the amount of food and hence, the maximum efficacy may have decreased in this study. Collectively, these findings suggest that SARM‐2f could have more potent anabolic effects in humans than in monkeys. To the best of our knowledge, this is the first report that demonstrates the anabolic action of SARM‐2f in monkeys.

Although the TE administration group showed a tendency for an increase in BW and LBM, it was not statistically significant. We were able to maintain the blood testosterone concentration necessary for clinical treatment of hypogonadism as described above. In additions, the EC_50_ values of SARM‐2f and testosterone were almost equal for monkey AR, and the AUC_0‐24h_ level was estimated to be higher for testosterone than SARM‐2f. Therefore, it is unlikely that the efficacy of TE was weak because of lower exposure of testosterone compared to SARM‐2f. Furthermore, it is reported that TE intramuscular administration at 20 mg/kg significantly increasing the BW of monkeys within 4 weeks, where the blood testosterone nadir levels is approximately 1500 ng/dL.^25^ This is much higher than the blood testosterone nadir levels in this study. Collectively, this study did not record a significant increase possibly because of the small sample size.

It should also be noted that different doses were used for TE and SARM‐2f in this study; ie 2 mg/kg im every 2 weeks for TE vs 1‐10 mg/kg p.o. daily for SARM‐2f. These administration routes for these drugs were chosen as per the best suitability of administration. TE was administered intramuscularly and we selected the dose of 2 mg/kg once every 2 weeks, mimicking the clinical regimen used in male hypogonadism (maximum 250 mg once every 2‐4 weeks; this dose is indicated on the package insert of TE). In contrast, SARM‐2f is a non‐steroidal small molecule that was optimized for oral administration. Since drug metabolism and hence the pharmacodynamics differ between TE and SARM‐2f, direct comparison of efficacy needs to be performed cautiously. The timing of efficacy measurements may affect the intensity and persistency of the drug effects.

We previously reported that SARM‐2f increased skeletal muscle weight with a much lesser effect on prostate weight.^15^, ^16^, ^17^ The mechanisms for tissue selectivity of SARM includes two factors: recruitment of specific cofactors to AR and a tissue‐specific cofactor expression profile.^26^, ^27^ We reported that SARM‐2f induced less recruitment of the protein inhibitors of the activated STATs family to the AR than did testosterone.^16^ The present study did not address the tissue specificity of SARM‐2f. Further research will be necessary to investigate its tissue specificity in monkeys.

The pharmacokinetic parameters were similar between rats and monkeys. The AUC_0‐24h_ values after a single oral administration of SARM‐2f at 1 mg/kg are 809.4 ng∙h/mL and 557.8 ng∙h/mL, respectively,^15^ and MRT (Mean Residence Time) was 3.06 and 5.21 hours, respectively, for rats and monkeys.^15^ The Vdss value after intravenous administration at 0.1 mg/kg was 2167 mL/kg and 1409 mL/kg for rats and monkeys, respectively.^15^ In this study, the effective dose to increase BW by approximately 10% in monkeys was 10 mg/kg, and the AUC_0‐24h_ at 10 mg/kg was 8152 ng·h/mL. A dose of 0.3 mg/kg is sufficient to increase BW in rats.^17^ These findings indicate that monkeys required an approximately 30‐fold higher drug exposure to gain BW, compared with rats.

In addition, 2 weeks or less of daily treatment with SARM‐2f is enough to increase BW in rodents.^16^ In contrast, BW was not significantly increased after 2 weeks of daily treatment with SARM‐2f at 10 mg/kg in this monkey study but was significantly increased at 4 weeks, suggesting that skeletal muscle increase in primates might require more time, as compared with rodents.

Monkeys were selected for the present study because we should to examine the effect of SARM‐2f on blood lipid profile. Rodent models are not suitable for pre‐clinical studies of lipid profiles because they differ from humans by having very low LDL‐c levels.^28^ Testosterone was reported to increase the blood LDL‐c level and reduce the blood HDL‐c level in patients with hypogonadism^29^, ^30^ and in sexually mature monkeys.^31^ In the present study, TE treatment increased the LDL‐c level but did not decrease the HDL‐c level in monkeys. This may be because the animals used in this study were not sexually mature. SARM‐2f decreased almost all of the lipid parameters measured in this study, including HDL‐c, LDL‐c, TG, and T‐chol. In the recent years, an increase in the LDL‐c/HDL‐c ratio, and increased LDL‐c levels, have been considered to represent risk factors for arteriosclerosis and myocardial infarction.^32^ Blood LDL‐c and HDL‐c levels were both decreased in monkeys treated with SARM‐2f, while the LDL‐c/HDL‐c ratio remained unchanged or decreased.

It is currently unclear whether the level of Lp (a), a potent risk factor for heart disease,^33^ is suppressed by statins, a widely used class of hydroxymethylglutaryl‐CoA reductase inhibitors.^34^, ^35^, ^36^ SARM‐2f showed a tendency to reduce blood Lp (a) levels in monkeys. Further studies are required to confirm this effect.

Blood AST and ALT levels, which provide an index of liver function, were not affected by SARM‐2f at 10 mg/kg in this study. Therefore, the changes in the blood lipid profile associated with SARM‐2f administration were not caused by liver function deterioration.

It should also be noted that in 126 biochemical activity assays (enzyme assays or radioligand binding assays) provided by MDS Pharma Service‐Taiwan Ltd., the only molecule that SARM‐2f affected at 10 µmol/L was AR (Table S5). Based on these findings, we believe that SARM‐2f has no severe off‐target effects.

Taken together, these findings indicated that SARM‐2f had fewer negative impacts on the lipid profile, as compared with testosterone. However, further studies are required to investigate the long‐term effects of SARM‐2f on lipid profiles in monkeys.

Several major international medical societies provide the guidelines for testosterone therapy for testosterone deficiency or hypogonadism.^37^ The consensus amongst the guidelines is that testosterone deficiency is defined based on both abnormal laboratory measurements and clinical signs. The laboratory thresholds vary amongst the organizations but are similar worldwide. The American Urological Association defines a low total testosterone as <300 ng/dL.^37^ Clinical signs include anemia, bone mineral density loss, male infertility, loss of muscle, increase in adiposity, and erectile dysfunction.^37^ The European Association of Urology, the International Society for Sexual Medicine, and the International Society for the Study of the Aging Male set a threshold of 350 ng/dL, and the British Society for Sexual Medicine set <345 ng/dL. The American Association of Clinical Endocrinologists declined to set a threshold but suggested that symptomatic men with testosterone levels <200 ng/dL may be potential candidates for testosterone therapy.^37^ In this study, it was necessary to use juvenile or elderly monkeys to mimic clinical settings where hypogonadism or sarcopenia patients have low blood testosterone levels. The reasons for choosing juvenile monkeys are as follows: (a) juvenile monkeys used in this study showed blood testosterone levels below the threshold defined as hypogonadism; (b) elderly monkeys have large individual differences in BW^38^, ^39^; (3）blood testosterone levels differ greatly among individuals from the age of 4 years under breeding conditions^36^; and (4) longer‐term drug administration, both in the case of SARM‐2f and TE, was required in pharmacological studies to show significant increases in BW and LBM in older rats compared to juvenile rats (in‐house data). These findings suggest that the treatment period required for pharmacological studies in elderly monkeys would be very long. Because of these reasons, we used juvenile monkeys.

Despite selecting the animals so carefully, one animal in the vehicle control group may have entered the sexual maturation phase because it showed increasing blood testosterone levels during the experimental period. Blood androgen levels increase during the sexual maturation period.^38^ Normal body growth of 14 male indoor‐bred cynomolgus monkeys has been reported.^38^, ^39^ The variation in BW increases with age, and 4‐year‐old male cynomolgus monkeys gain approximately 0.7 kg in half a year.^38^, ^40^ This finding is consistent with our data in this study, where the vehicle treatment monkeys showed an increase of 0.1 kg in 4 weeks. Because of the animal in the vehicle group, it was slightly difficult to demonstrate the anabolic effects of SARM‐2f.

One of the limitations of this study is that the responses to SARM may differ between immature animals with low testosterone levels and older animals with low testosterone levels. Further study will be necessary to investigate the effects of SARM‐2f on older monkeys with low blood testosterone levels.

In elderly people or patients with metabolic wasting diseases, the ability to absorb protein and vitamins deteriorates, leading to muscle loss.^41^, ^42^ Comprehensive therapeutic strategies combining exercise, nutritional control, and SARM treatment may therefore provide an effective approach to strengthening muscle power in patients with sarcopenia or cachexia. In conclusion, SARM‐2f demonstrated anabolic effects and hypolipidemic activity in monkeys. These findings warrant further study to explore whether SARM‐2f could be used in humans.

## CONFLICTS OF INTEREST STATEMENT

The authors have no conflict of interest to the content of this article.

## AUTHOR CONTRIBUTIONS

Morimoto, Yamaoka, and Hara participated in research design and conducted experiments*.* Morimoto performed data analysis*.* Morimoto and Hara contributed to the writing of the manuscript*.*
