# Supplementary material for: A selective androgen receptor modulator SARM‐2f activates androgen receptor, increases lean body mass, and suppresses blood lipid levels in cynomolgus monkeys
Source: Pharmacol Res Perspect. 2020 Feb 7;8(1):e00563. doi: 10.1002/prp2.563 (PMC7005530; doi:10.1002/prp2.563)
Supplement: Supplementary file 1 [file PRP2-8-e00563-s001.docx]

**Supplemental Figure 1**

Plasma pharmacokinetics of SARM-2f in monkeys.

Concentrations of SARM-2f in plasma after a single- or 14-times oral dosing at 10 mg/kg in 2 male and 2 female monkeys.


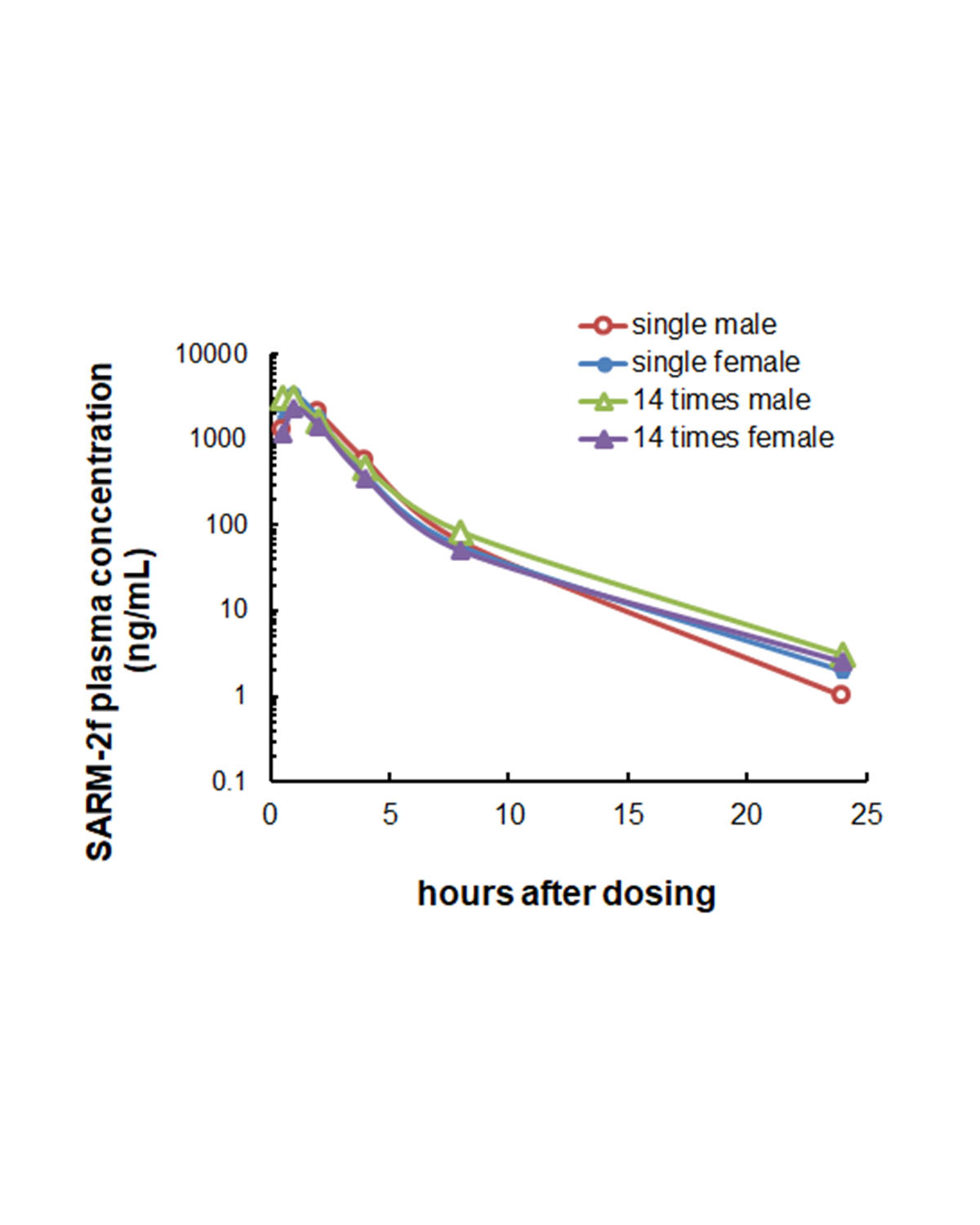


Supplemental Table 1

Supplemental Table 2

Supplemental Table 3

Supplemental Table 4

Food of 108g was served daily for each monkey, and the amount of food consumption was measured every day.

Supplemental Table5
